# Extracellular Vesicle (EV) Array: microarray capturing of exosomes and other extracellular vesicles for multiplexed phenotyping

**DOI:** 10.3402/jev.v2i0.20920

**Published:** 2013-06-18

**Authors:** Malene Jørgensen, Rikke Bæk, Shona Pedersen, Evo K.L. Søndergaard, Søren R. Kristensen, Kim Varming

**Affiliations:** 1 Department of Clinical Immunology, Aalborg University Hospital, Aalborg, Denmark; 2 Department of Biochemical Chemistry, Aalborg University Hospital, Aalborg, Denmark

**Keywords:** EV Array, exosomes, extracellular vesicles, phenotyping, antigenic capturing, nanoparticle tracking analysis, protein microarray, plasma

## Abstract

**Background:**

Exosomes are one of the several types of cell-derived vesicles with a diameter of 30–100 nm. These extracellular vesicles are recognized as potential markers of human diseases such as cancer. However, their use in diagnostic tests requires an objective and high-throughput method to define their phenotype and determine their concentration in biological fluids. To identify circulating as well as cell culture-derived vesicles, the current standard is immunoblotting or a flow cytometrical analysis for specific proteins, both of which requires large amounts of purified vesicles.

**Methods:**

Based on the technology of protein microarray, we hereby present a highly sensitive Extracellular Vesicle (EV) Array capable of detecting and phenotyping exosomes and other extracellular vesicles from unpurified starting material in a high-throughput manner. To only detect the exosomes captured on the EV Array, a cocktail of antibodies against the tetraspanins CD9, CD63 and CD81 was used. These antibodies were selected to ensure that all exosomes captured are detected, and concomitantly excluding the detection of other types of microvesicles.

**Results:**

The limit of detection (LOD) was determined on exosomes derived from the colon cancer cell line LS180. It clarified that supernatant from only approximately 10^4^ cells was needed to obtain signals or that only 2.5×10^4^ exosomes were required for each microarray spot (~1 nL). Phenotyping was performed on plasma (1–10 µL) from 7 healthy donors, which were applied to the EV Array with a panel of antibodies against 21 different cellular surface antigens and cancer antigens. For each donor, there was considerable heterogeneity in the expression levels of individual markers. The protein profiles of the exosomes (defined as positive for CD9, CD63 and CD81) revealed that only the expression level of CD9 and CD81 was approximately equal in the 7 donors. This implies questioning the use of CD63 as a standard exosomal marker since the expression level of this tetraspanin was considerably lower.

Tumour cells and cells in general release membranous structures, which are termed microvesicles or exosomes depending on specific characteristics, including size and composition (1). Microvesicles and exosomes, in particular, have recently gained paramount attention as “vehicles” for intercellular communication with extensive autocrine/paracrine functions. By exposing cell-type-specific adhesion receptors or ligands, exosomes can interact with specific cells and deliver their “signals” including bioactive lipids, cytokines, growth factors, receptors and genetic material (2, 3). Thus, the microvesicle/exosomal pathway may constitute a mechanism for local and systemic intercellular transfer of information with a complexity superior to that of secreted soluble factors, but similar to that observed with direct cell–cell contact (4).

Exosomes have been defined based on size (30–100 nm lipid bilayer vesicles), density (1.12–1.19 g/ml) and expression of specific biomarkers (e.g. tetraspanins, annexins and heat shock proteins) (5). The direct use of circulating cell-derived vesicles for disease diagnosis has been limited by the current lack of methods to measure and characterize these. The protein composition of the vesicles can be determined by flow cytometry and immunoblotting, but neither of these analyses provide information on the total number of vesicles nor the overall distribution of subtypes.

Our goal is to investigate whether an Extracellular Vesicle (EV) Array using antigenic capturing of exosomes or other extracellular vesicles by protein microarray could be used to establish an authentic reflection of both phenotype as well as enumeration of circulating vesicles. This is exemplified with the capture of exosomes, which in the following are defined as vesicles carrying CD9, CD63 and/or CD81. Protein microarrays are well accepted as powerful tools to search for antigens or antibodies in various sample types (6, 7). It is used as a high-throughput method to track the interactions and activities of proteins on a large scale. The superiority of protein microarray is that large numbers of proteins can be tracked in parallel; it is a rapid, automated, economical and highly sensitive method consuming only small quantities of samples and reagents.

This study demonstrates the potential of the EV Array for estimating the content and distribution of vesicles in clinical plasma samples, as well as in samples with cell culture-derived exosomes and other extracellular vesicles. While cell-derived vesicles can be detected in the blood circulation and other body fluids, they are a blend of vesicles derived from many different cell types including platelets, endothelial cells, leukocytes and red blood cells, which complicate their analysis. Furthermore, the ability of the EV Array to generate a multiplexed phenotyping for these vesicles was also investigated.

## Materials and methods

### Cell cultures

The cancer cell lines SW948, LS180 and OAW42 were cultured in RPMI-1640 (Fisher Scientific, USA) supplemented with 10% heat-inactivated foetal calf serum (FCS, Life Technologies, CA, USA) centrifuged at 100,000×*g* for 16 h, 100 U/mL penicillin and 0.1 mg/mL streptomycin (both VWR, PA, USA) at 37°C in 5% (v/v) CO_2_ air atmosphere.

### Preparation of exosomes from cell cultures

SW948 and OAW42 cells (80 cm^2^ flasks, VWR) at 80–90% confluence were washed twice with phosphate-buffered saline (PBS) and then incubated in fresh medium for 24 h. Approximately 45 mL of conditioned medium was collected, centrifuged at 500×*g* for 10 min and then filtered (0.22 µm) prior to the addition of protease inhibitors (Complete, EDTA-free, Roche, DE, USA). The medium was concentrated using a 100K MWCO spin filter (Amicon, Merck Millipore, MA, USA) and the concentrate was washed 3 times in PBS and stored at −40°C. The exosome-containing media was concentrated approximately 100 times.

LS180 cells were cultured in microtitre trays in a range from 7×10^2^ to 1×10^5^ cells per well in 200 µL culture media for 48 h. Non-adherent cells were pelleted by centrifugation of the microtitre tray for 10 min at 3,200×*g* and the resulting supernatant was harvested and protease inhibitors were added prior to analysis or storage at −40°C.

### Blood samples

Blood samples were obtained from healthy blood donors at the Department of Clinical Immunology at Aalborg University Hospital as part of the Danish Blood Donor Study (www.dbds.dk). Blood samples were collected in citrate (S-Monovette, Sarstedt, DE, USA) and centrifuged at 3,000×*g* for 6 min to sediment cells. The plasma was removed, aliquoted and stored at −40°C until analysis.

## EV Array

### Production of microarray

Microarray printing was performed on a TopSpot E-vision non-contact printer with a 24-spot print head (Biofluidix GmBH, Freiburg, DE, USA). As positive and negative controls, 100 µg/mL of biotinylated human IgG and PBS with 5% glycerol was printed, respectively. Epoxy-coated slides (75.6 mm×25.0 mm, SCHOTT Nexterion, DE, USA) were used and then left to dry at room temperature overnight prior to further analysis.

### Antibody setup for phenotyping

The antibodies were printed at 87.5–400 µg/mL diluted in PBS with 5% glycerol. The chosen antibodies against human antigens were: tumour necrosis factor receptor (TNF R) I and TNF RII (R&D Systems, MN, USA); epithelial cell adhesion molecule (EpCAM, clone 0.N.277), cancer/testis antigen 1 (CTAG1, NY-ESO-1, clone E978), placental alkaline phosphatase (PLAP, clone 8B6), coilin (clone F-7), glucose-regulated protein 78 (GRP78, clone N-20) and mucin16 (clone X306) (Santa Cruz Biotechnology, CA, USA); CD276 (Sdix, DE, USA); surfactant protein D (SFTPD, clone VIF11) and osteopontin (Acris, DE, USA); heat shock protein 90 (Hsp90, clone IGF1) and p53 (clone pAb240) (Abcam, Cambridge, UK); epidermal growth factor receptor (EGFR) (Antibodies-online.com, GA, USA); surfactant protein A (SPA, clone 6F10) (Novus Biological, CO, USA); Paired Box-8 (PAX-8) (Cell Marque, CA, USA); human epidermal growth factor receptor 2 (HER2/ErbB2, Clone 29D8) (Cell Signaling Technology, MA, USA); CD9 and CD81 (LifeSpan Biosciences, Inc., WA, USA); CD63 (Clone MEM-259) and HLA-ABC (Clone W6/32) (BioLegend, CA, USA).

### Antibody setup for quantification (cocktail slide)

The antibodies were printed in a mixture/cocktail of 100 µg/mL of each antibody diluted in PBS with 5% glycerol. The antibodies against human antigens were: CD9 and CD81 (LifeSpan Biosciences, Inc., WA, USA); CD63 (Clone MEM-259) (BioLegend, CA, USA).

### Catching and visualization

The dried printed slides were blocked with 50 mM ethanolamine, 0.1 M Trisbase and 0.1% SDS, pH 9.0 for 1 h prior to insertion into multi-well cassettes (ArrayIt, CA, USA) for 24 samples per slide. Samples were applied after being diluted in washing buffer (PBS, 0.2% Tween^®^20), whereafter they were incubated with mild agitation at 2 h at room temperature following 18 h at 4°C.

After incubation, the slides were removed from the multi-well cassettes and washed in washing buffer for 10 min in a high-throughput washing station (ArrayIt). A cocktail of biotinylated detection antibodies diluted in PBS 1:1,500 (anti-human-CD9, CD63 and CD81, LifeSpan BioSciences, Inc., WA, USA) were applied and incubated with mild agitation for 2 h at room temperature.

After incubation, the slides were washed for 10 min prior to the addition of streptavidin-Cy5 (Life Technologies) diluted in PBS 1:1,500. After 30 min of incubation at room temperature with mild agitation, the slides were washed for 10 min followed by a 10 min wash in MilliQ water. Prior to scanning, the slides were dried with compressed air.

### Elution from EV Array

After sample incubation and washing in washing buffer for 10 min, the slides were once again inserted into the cleaned multi-well cassettes. Hundred microlitres of different elution buffers was added (100 mM glycine-HCl, pH 2.4; 100 mM glycine-HCl, 0.5 M NaCl, pH 2.4; 100 mM glycine-HCl, 0.5 M NaCl, 0.05% Tween^®^20, pH 2.4; 100 mM glycine-HCl, 0.05% Tween^®^20, pH 2.4; IgG elution buffer (ThermoScientific, USA); or 200 mM NaOH pH 10.5) and incubated on an orbital shaker for 30, 60, 120, 240 or 1,200 min. After removal of the eluted exosomes, the wells were washed 3 times in washing buffer to stop further elution prior to incubation with detection antibodies and streptavidin-Cy5, as described earlier. The eluted samples were neutralized by the addition of 1 M Tris.

### Scanning of microarray slides

Scanning was performed on an Innoscan 710AL scanner (Innopsys, Carbonne, France) with a 635 nm laser and the settings: 60% PMT, 10 mW, scan speed at 35 lines per second and a resolution at 5 µm. Images were analyzed using Mapix ver. 6.3.0 (Innopsys, Carbonne, France) and spots were automatically detected and then verified manually. The total intensity of the spots was calculated and exported for data analysis in Excel ver. 2007 (Microsoft, USA).

## Nanoparticle Tracking Analysis

### NanoSight analysis

The diameters of the microvesicles were measured using a NanoSight LM10-HS system equipped with a finely tuned 405 nm laser (NanoSight Ltd., Amesbury, UK). Samples were measured in the “single shutter and gain” mode for 60 s. A temperature-measuring device inserted directly into the sample chamber was applied to record the temperature of sample for each run. The Nanoparticle Tracking Analysis (NTA) 2.3 analytical software version was used for capturing and analysing the data.

Each experiment was carried out in triplicate. The NanoSight was calibrated with polystyrene latex microbeads at 50 nm, 100 nm, and 200 nm (Thermo Scientific, Fremont, USA) prior to analysis. Dulbecco's phosphate buffered saline (DPBS) without Ca^2 +^ and Mg^+^ (Lonza, Verviers, Belgium) was used to dilute the microbeads and starting material (plasma or exosomal elutions; 1:1,000 dilution).

### Statistical analysis

The mode particle size, the value of the highest point of the peak and standard deviation (SD) values obtained by the NTA software were used to describe the results. Mean results were calculated for each size. SD was found and the coefficient variation (CV%) for each size was calculated by the SD in relation to the mean particle size. All statistics were evaluated using Microsoft Excel ver. 2007. Each experiment was carried out in triplicate.

### Data analysis

Graphs and statistics were performed in GraphPad Prism ver. 5.04 (GraphPad software, Inc., CA, USA) and hierarchical clustering analysis (HCL, complete linkage clustering) was performed in Genesis ver. 1.7.6 (IGB TU Graz, Austria).

For each protein spot, the signal intensity was calculated by subtracting the median of the foreground from the median of the background (no sample, PBS) at 635 nm. For a given antibody spot, the signal intensity was calculated as the mean signal of duplicate spots in relation to the sample signal of a negative spot (PBS). Each data point was calculated as mean and SD of minimum 3 replicates. Before clustering analysis, the antibody signals were converted into log2 transformation.

## Results

### Protein microarray technology for extracellular vesicle detection (EV Array)

**Fig. 1:**
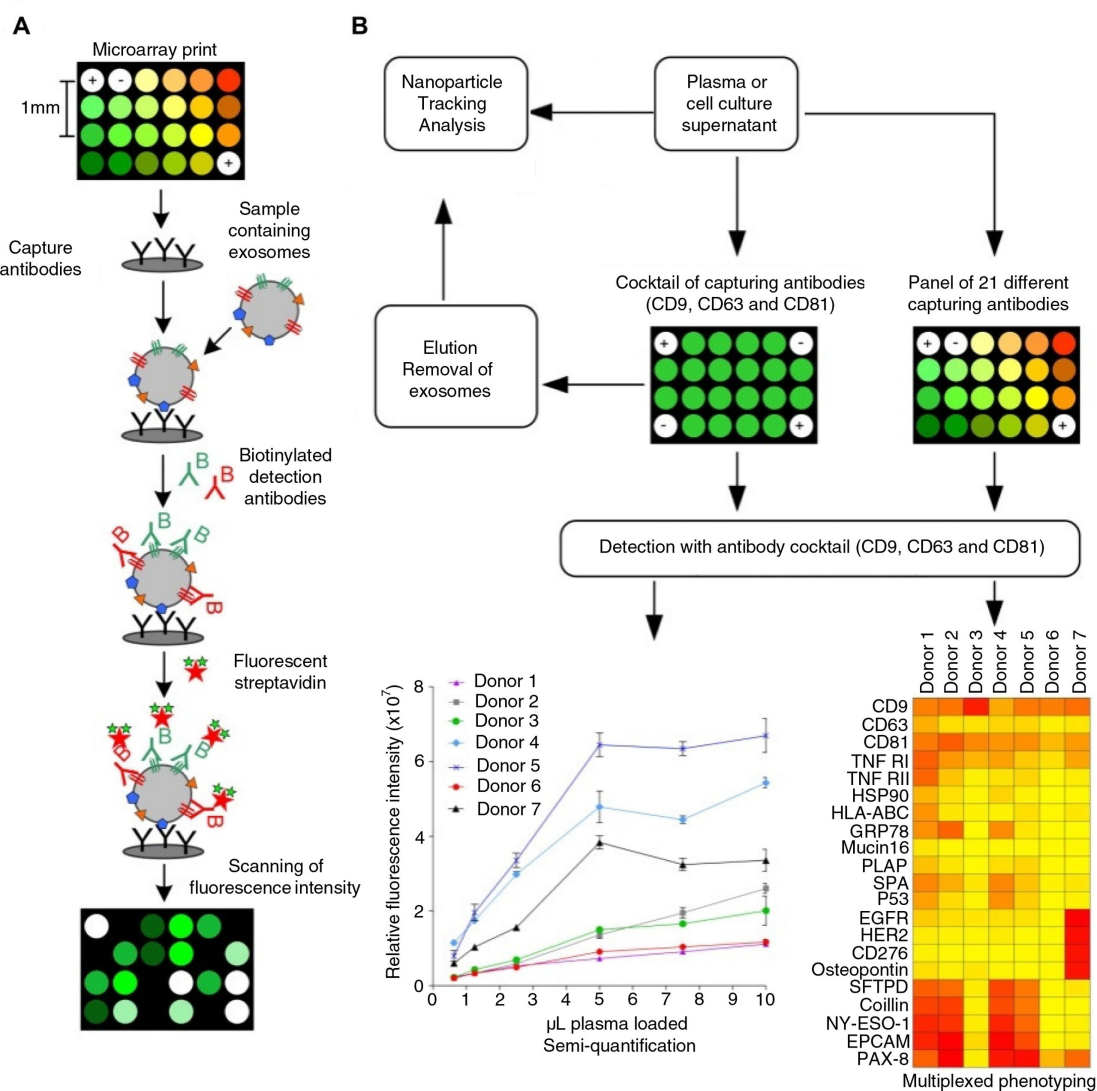
(A) Extracellular vesicle detection using a customized protein microarray “EV Array”. A microarray print with spots of 21 different antibodies is used to capture exosomes from, for example, plasma or cell culture supernatants. The captured vesicles (exosomes) are detected with a cocktail of biotinylated antibodies against the tetraspanins CD9, CD63 and CD81 followed by fluorescence-labelled streptavidin. (B) The technology of using protein microarray for exosome detection was validated using NTA and a series of experiments with different exosome sources and microarray print setups as illustrated in the flowchart. Microarray print with a cocktail of the capturing antibodies CD9, CD63 and CD81 was used to detect the signals from all exosomes present in the samples in a semi-quantitative manner in order to obtain the limit of detection (LOD, Fig. 3). This setup was also used to capture exosomes prior to elution (supplemental material) and validation by NTA. Microarray prints with a panel of 21 different capturing antibodies was used to phenotype the exosomes (positive for CD9, CD63 and/or CD81) present in plasma from 7 healthy individuals (Fig. 4).

The technology of protein microarray was used to capture exosomes and other extracellular vesicles from plasma and cell culture supernatants. Spots of capturing antibodies against known vesicular surface antigens were printed in a customized 24-spot setup using a non-contact printing technology (Fig. 1A). The EV Array (21 antibody spots; 2 positive controls; 1 negative control) was printed on standard epoxysilane-coated microarray slides in patterns fitting a duplicate of the print in each well of a multi-well cassette. The use of the 96-well multi-well cassettes allowed the EV Array to be performed as a high-throughput procedure using a minimum of sample.

Up to 100 µL of vesicle-containing sample can be applied to each well. The captured vesicles are detected using a cocktail of biotinylated antibodies against the tetraspanins CD9, CD63 and CD81, known as antigens present on exosomes in general (8–10). These antigens are used in the following to exemplify the use of the EV Array to detect only exosomes in a pool of extracellular vesicles. Using this technology, exosome detection and phenotyping was validated in a series of experiments (Fig. 1B).

In order to verify that exosomes are captured on the microarray slide, samples of cell culture supernatants and plasma were applied, and a series of elution experiments were performed (Supplemental Figs. S1 and S2). The most optimal elution was seen using 100 mM of glycine-HCl, pH 2.4 for 30 min, where approximately 90% of the exosomes were eluted. In order to obtain a maximal and effective capture of exosomes and to set the limit of detection (LOD), an array of 40 identical spots containing a cocktail of antibodies against CD9, CD63 and CD81 was generated and used for either elution or quantification.

**Fig. 2:**
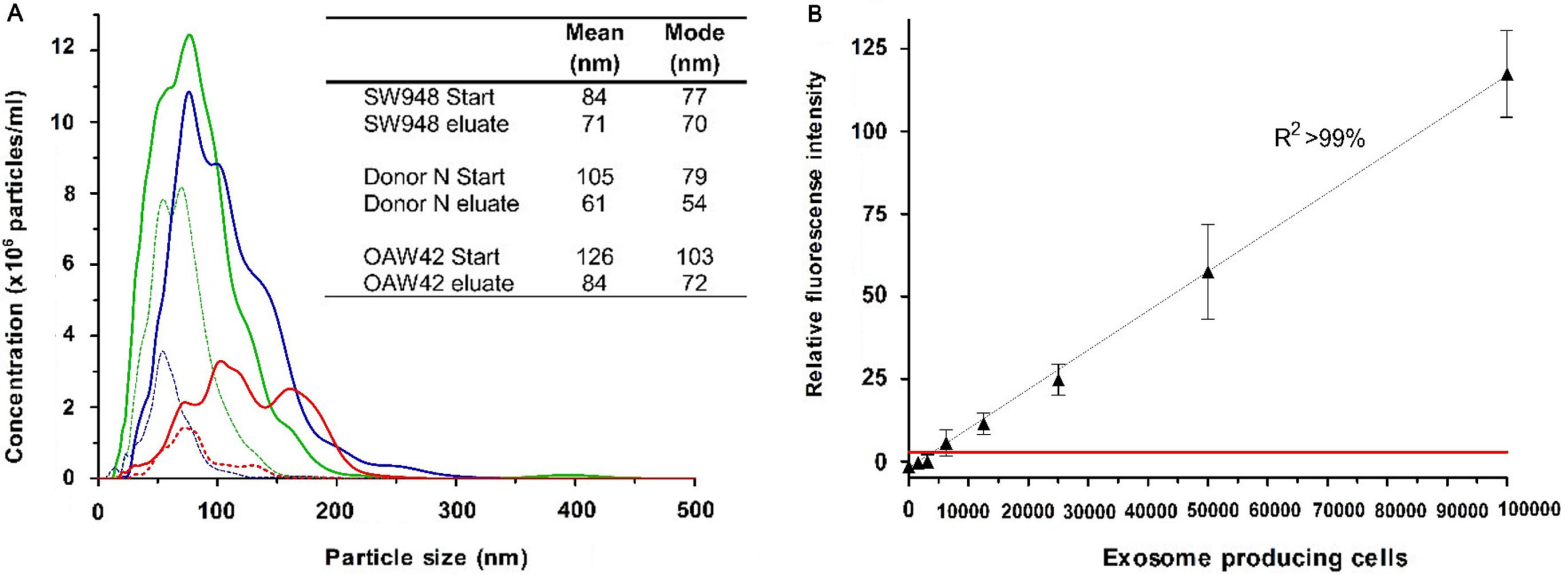
(A) Nanoparticle Tracking Analysis of the starting material from the EV Array analysis compared to the eluted particles after capturing on a microarray printed with a cocktail of antibodies against CD9, CD63 and CD81. The analyses were performed on a plasma sample (Donor N) and on cell media containing exosomes from the cancer cell lines SW948 (colon) and OAW42 (ovarian). No signals were obtained above 500 nm. The mode and mean values for each sample are given. (B) EV Array detection of exosomes captured on slides printed with a cocktail of antibodies against CD9, CD63 and CD81. The fluorescence intensities (mean±SD) are shown in relation to the number of LS180 (colon cancer) cells producing the exosomes within 48 h. A linear correlation (*R* ^2^>99%) is seen and the red line indicates 2 SD of the negative control demonstrating a limit of detection (LOD).

NTA was performed on both starting material and eluted exosomes (Fig. 2A). The NTA showed that the starting materials contained various amounts of exosomes and other microvesicles ranging from 0 to 300 nm in size. However, the samples eluted from the EV Array mainly contained vesicles with a size >100 nm indicating the array to be highly specific to exosomes. This is also seen by the lower mean and mode values when comparing the samples of starting material and the eluted samples.

The NTA indicated a lower level of eluted exosomes in relation to the starting material, which could be caused by saturation of the printed antibodies (and by losses in the experimental handling). Alternatively, it could be due to protein aggregation generated in the spin-filter during pre-treatment of the samples that could appear as small vesicles in the NTA of the starting material. However, it was also observed that exosomes eluted with a low pH seemed to have undergone some physiological changes impacting their Brownian motions and thereby limiting their detection by NTA (data not shown).

### Limit of detection

To test the LOD of the EV Array, a series of dilution experiments were performed with both the cell culture supernatants and human plasma. Figure 2B shows a linear correlation of the fluorescence signal obtained from exosomes captured on the microarray to the number of cells producing the exosomes. Each point reflects the signal from exosomes captured directly from a non-purified cell culture supernatant harvested from a microtitre well with a defined number of cells. The LOD using cell culture supernatants is for the cell line LS180 exosomes produced by only 1.25×10^4^ cells under the given conditions.

**Fig. 3:**
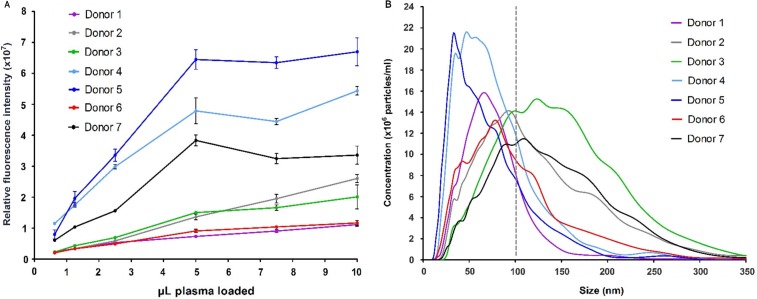
Microvesicle analysis of plasma from 7 healthy donors. (A) A series of dilutions were tested in triplicates on EV Array slides printed with a cocktail of capturing antibodies (against CD9, CD63 and CD81). The relative fluorescence intensities of the spots (mean±SD) are plotted against the volume of plasma added. (B) Nanoparticle Tracking Analysis of the plasma revealed a great variance of the microvesicle distribution and particle concentration. Donors 4 and 5 are observed to have a higher number of vesicles <100 nm (exosomes) as indicated by the grey line.

In the literature, microparticle concentrations in plasma range from 10^7^ to 10^12^ L^−1^ (11–14). To deal with this gap in concentration, we tested plasma from 7 healthy donors in a series of dilutions. Exosomes from non-purified plasma (0.1–10 µL) were captured and detected on a microarray printed with a cocktail of antibodies against CD9, CD63 and CD81. The detected fluorescence signal revealed a linear relation until the spots are saturated (Fig. 3A). Saturation of the spots is seen with >5 µL plasma for donors 4, 5 and 7. NTA of the plasma samples showed a great variation in the microvesicle content of the 7 donors (Fig. 3B and Fig. S3A and B), where donors 4 and 5 were significantly different having a content of vesicles/exosomes mainly with a size <100 nm. This correlates with the data obtained from the EV Array (Fig. 3A) although a full quantitative correlation between the methods was not expected (and not tested), as the EV Array depends on CD9, CD63 and CD81 to be equally expressed in all exosomes as well as in all individuals to gain a fully quantitative measurement.

The LOD for exosomes derived from plasma is unattainable, as plasma tends to impart a sample-specific background on the microarray. The exosomes were clearly detectable by using only 1 µL of plasma. However, for semi-quantification 5 µL is optimal in regard to the signal-to-noise ratio. A comparison of the quantification of microvesicles >100 nm (NTA) to the microarray data reveals that only 1×10^6^ exosomes are needed for a significant signal. This signal was measured on 40 replicates of the antibody cocktail spot determining each spot to capture 2.5×10^4^ exosomes.

### Phenotyping of plasma-derived exosomes

Techniques such as NTA and dynamic light scattering (DLS) are limited to objectively define the microvesicle size range and to estimate microvesicle concentration. NTA can be extended to detect fluorescence-labelled microvesicles to phenotype a single antigen per measurement (15). In addition, phenotyping by NTA is hindered by laser capacity.

Using microarray printed with cell-type-specific antibodies, it is possible to identify specific subsets of microvesicles as, for example, exosomes. Plasma from 7 healthy donors was applied to a panel of antibodies against 21 different cellular surface antigens and cancer antigens. As seen in Fig. 3A, the total level of microvesicles in the plasma samples differed considerably, therefore a log2 transformation was performed prior to cluster analysis.

**Fig. 4:**
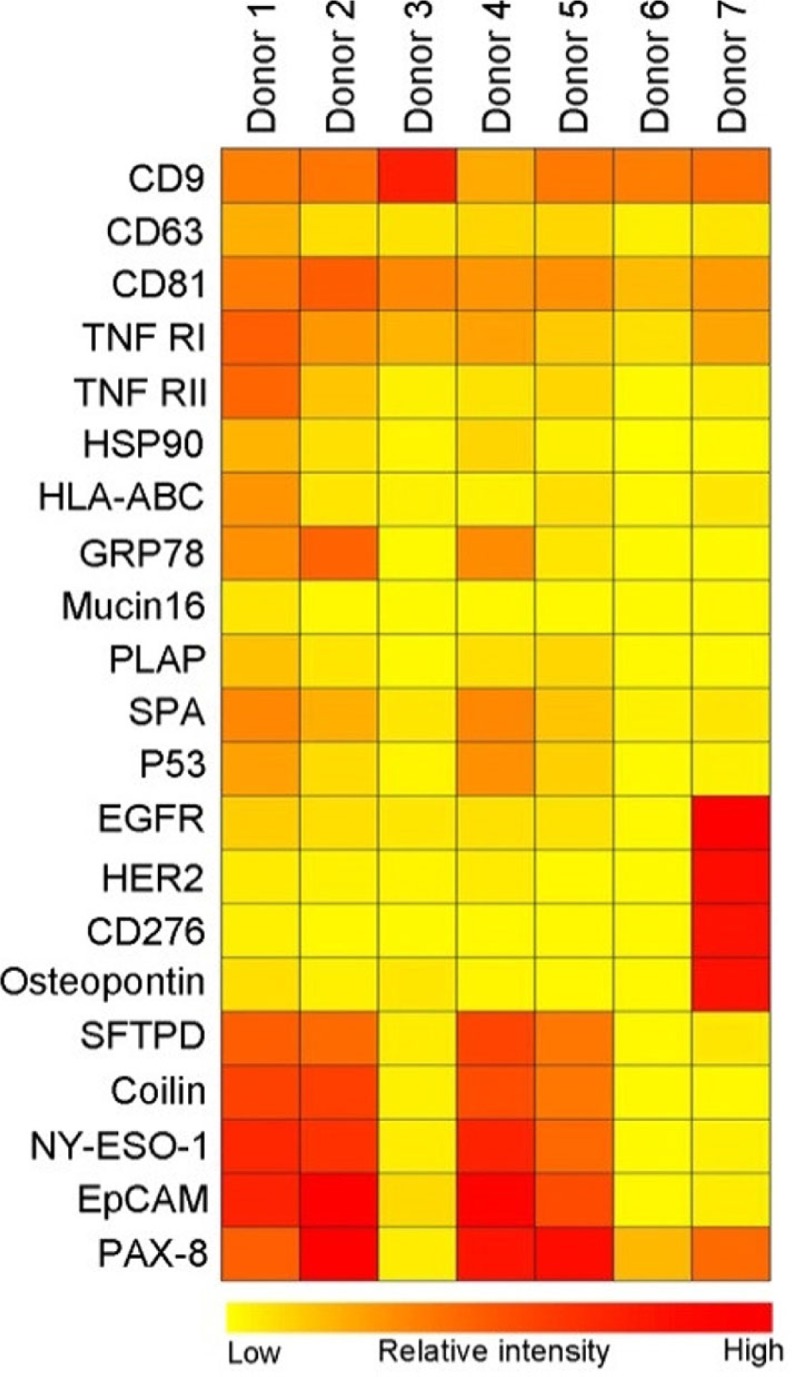
Summary of the phenotyping of the exosomal (positive for CD9, CD63 and/or CD81) population in plasma from 7 healthy donors. The exosomes were profiled using an EV Array printed with 21 different capturing antibodies. The relative fluorescence intensity was log2 transformed and a hierarchical clustering was performed to illustrate the phenotypes of the plasma-derived exosomes.

For each donor, there was considerable heterogeneity in the expression levels of individual markers (Fig. 4). The protein profiles of the exosomes revealed the fact that the tetraspanins CD9 and CD81 were found to be expressed at approximately equal levels in the 7 donors. In this relation, a lower level of CD63 was generally observed indicating either a lower number of exosomes expressing CD63 or a lower level of CD63 present on each exosome. As several antibodies against CD63 were tested with similar results (data not shown), these observations cannot be explained by differences in antibody specificity.

Donors 4 and 5, having high amounts of exosomes, showed an exosomal phenotype similar to donors 1 and 2 indicating that a high concentration of exosomes does not seem to influence the protein composition of the exosomes. However, plasma from donor 7 showed a very distinct phenotype of exosomes carrying higher levels of EGFR, HER2, CD276 and osteopontin; all known cancer antigens. The exosomal phenotype of donors 3 and 6, with low concentration of exosomes, mainly showed expression of the general exosomal markers, CD9 and CD81 but also TNF RI.

## Discussion

Exosomes are 30–100 nm membrane vesicles of endocytic origin secreted by most cell types *in vitro*. Studies have shown that exosomes are also found *in vivo* in body fluids, such as blood, urine, amniotic fluid, malignant ascites, bronchoalveolar lavage fluid, synovial fluid and breast milk. While the biological function of exosomes remains unclear, they are known to mediate communication between cells and facilitate processes such as antigen presentation as well as in trans signalling to neighbouring cells [reviewed by Théry et al. (16)]. These cell-derived exosomes are potential markers of human diseases. However, their use in diagnostic tests requires an objective and high-throughput method for defining their concentration and phenotype in biological fluids. Current technologies cannot achieve this definition and characterization. The results of this study demonstrate the feasibility of using protein microarray. Using plasma from healthy donors, we demonstrate that protein microarray can capture and estimate the concentration of cell-derived vesicles of exosomal character. Hereby, we have developed a new application for protein microarray, an EV Array.

NTA is a relatively new technique for analysis, detection and visualization of individual vesicles in real time (15, 17). A significant limitation of NTA though is that while it can objectively define the vesicle size range and concentration, it is restricted in its ability to phenotype vesicles using several antigens simultaneously. In this study, we compared the results from protein microarray analysis with the results from NTA to define the size distribution and number of circulating cell-derived vesicles from healthy donors. Using NTA to validate the detection of microvesicles, we demonstrated that the vesicles captured with an antibody mixture against CD9, CD63 and CD81 and analyzed by microarray have a size below 100 nm indicating an exosomal origin. Therefore, we have taken the liberty of denoting our captured vesicles as exosomes although we are well aware that there might be traces of other types of microvesicles present.

To define the identity of circulating as well as cell culture-derived vesicles, the current “gold standard” is immunoblotting or a flow cytometrical analysis for specific marker proteins. Conventional flow cytometers do not allow detection of particles <300 nm based on forward scattered light (FSC). Therefore, antibody-coated latex beads (4 µm) are used to capture and carry the vesicles into the flow cytometer (18). Van der Vlist et al. developed a new type of flow cytometry-based method for analysis of approximately 100 nm fluorescent particles (19). This type of high-resolution flow cytometric analysis allows the simultaneous analysis of multiple parameters on single vesicles. For a single analysis on T-cell-derived microvesicles, they used vesicles produced by 1×10^7^ cells. A multicolour labelling strategy allowed them to demonstrate large differences in the protein composition of vesicle populations derived from lipopolysaccharide-activated and non-activated dendritic cells (19). A standard analysis for a single antigen using conventional flow cytometry and western blotting demands 10–30 µg of exosomal or extracellular vesicular protein isolated by extensive and time-consuming standard isolation procedures (10). In order to obtain that amount of purified exosomal material, large quantities of cells producing the exosomes are needed (3×10^7^–4×10^8^ cells). In the literature, various cell types with various growth conditions have been used for exosome purification with different yields (20–26).

Using our EV Array printed with a cocktail of antibodies against CD9, CD63 and CD81, we demonstrated that it was possible to capture and detect exosomes in supernatant from as little as approximately 10^4^ LS180 cells. Even more importantly, this was performed without any time-consuming isolation or enrichment of the exosomes prior to analysis. Our method makes it possible to semi-quantify and phenotype exosomes directly from small cell cultures. In perspective, this EV Array technology makes it possible to setup large-scale experiments monitoring effects of various cell stimulations and conditions on the production of exosomes or other extracellular vesicles.

Since biological fluids and clinical specimens are composed of a mixture of vesicles derived from many cell types, it is essential to be able to determine the cell origin and to understand their biological function. The ultimate and critical application of exosome-based diagnostic markers is the ability to identify the presence of circulating cell-derived vesicles and their concentration in clinical specimens. In this study, we determined the presence and distribution of circulating exosomes (positive for CD9, CD63 and/or CD81) in plasma from healthy controls and cell culture supernatants. Similar approaches and analytical techniques for exosome protein typing have been developed: Using the NanoSight LM10 equipped with a 405 nm blue-violet laser, Gercel-Taylor et al. demonstrated that antibodies against either CD63 or EpCAM labelled with quantum dots could be used to phenotype exosomes purified from patients with ovarian cancer (27). Shao et al. developed a miniaturized micro-nuclear magnetic resonance (µNMR) system for protein typing of microvesicles with a detection threshold of approximately 10^4^ vesicles which is comparable to this study (28). However, this µNMR system can only detect the presence of a single exosomal antigen at a time (as for flow cytometry). In contrast to microarray detection, both of these techniques rely on an enrichment or purification of the exosomes prior to analysis.

Currently, no proteins are known to be constitutively sorted into vesicles independently of the subcellular origin of the vesicle and the activation status of the producing cell. This lack of invariant “household” markers hampers the quantitative analysis of vesicles. Peters et al. discovered that exosomes derived from B cells contained the lysosomal membrane protein CD63 (8) and later on several other members of the tetraspan superfamily were detected to be enriched in exosomes (29). These early studies also showed that CD63 are found to be under-represented in exosomes in relation to the concentration found in the producing cells. Whereas CD81 was found to be more than 10-fold up-regulated in exosomes (29). The phenotyping of plasma exosomes from 7 healthy donors revealed a similar tendency showing CD63 to be less represented in relation to CD81 and CD9. This could open discussions for; whether CD63 is the optimal exosomal marker or if it is just the marker first discovered and described and therefore used in general (8).

To detect the exosomes captured on the EV Array, a cocktail of antibodies against CD9, CD63 and CD81 was used. These antibodies were selected to ensure that all exosomes captured would be detected, as well as excluding other types of microvesicles to be detected. The developed technology of EV Array leaves the possibilities open to simply change these detection antibodies in order to detect other populations or sub-populations of extracellular vesicles as, for example, tissue-factor bearing vesicles. Hereby, we have generated an open platform, which is easy to transform and develop further if future scientific experiments reveal other exosomal or extracellular markers.

For plasma, the EV Array could be used to distinguish, for example, exosomes produced by leucocytes in general or T-cells specifically, or from cell lines such as LIM1863 which is known to produce more than one type of exosomes (24). Depending on scanning possibilities, the sub-types of the phenotypes could also be detected simultaneously using a dual-, or triple-colour detection. This could generate datasets reflecting both the phenotype of the total pool of exosomes present together with a specific phenotype of the exosomes produced by specific cells, as, for example, cancer or immune cells.

The EV Array used to develop and verify this technique contained 21 different antibodies to capture the exosomes. The number of antibodies used in multiplex phenotyping is only limited by the microarray printing technology (24-well print head for this non-contact printing) and can be expanded with additional printing procedures or other printing technologies. This also opens the opportunities to develop a customized panel of capturing antibodies. The phenotyping of plasma-derived exosomes from healthy individuals revealed a great variance in the protein profiles of the exosomes regardless of their actual quantity. Since the biological function of the exosomes is still unclear and extensively being investigated, this variance in the exosomal phenotypes present in plasma illustrates the needs for more studies of healthy individuals. For this, technologies such as the EV Array for multiplexed phenotyping are essential.

While this study focused on healthy donors, the technique could also be used for tumour-specific exosome tracking. It is, however, essential to recognize that any specific marker may not be expressed on all tumours, their expression is likely to be heterogeneous within the primary tumour, and their expression may be different between primary and metastatic tumours within the same individual. The potential of using exosomes as diagnostic markers for cancer, rheumatisms or other diseases demands technologies, such as the EV Array that necessitates the possibilities to detect exosomes in a highly sensitive, multiplexed and high-throughput manner without extensive isolation procedures.
